# Dual-Pathway Antithrombotic Therapy in Patients With Atrial Fibrillation After Percutaneous Coronary Intervention in Stable Coronary Artery Disease: A Single-Center, Single-Operator, Retrospective Cohort Study

**DOI:** 10.3389/fmed.2020.00414

**Published:** 2020-09-30

**Authors:** Lukas Andreas Heger, Martin Danzer, Christoph Bode, Marcus Hortmann, Daniel Duerschmied, Christoph B. Olivier, Martin Moser

**Affiliations:** Department of Cardiology and Angiology I, Heart Center Freiburg University, Faculty of Medicine, University of Freiburg, Freiburg, Germany

**Keywords:** atrial fibrillation, percutaneous coronary intervention, antithrombotic therapy, triple therapy, oral anticoagulation, single center, single operator retrospective analysis

## Abstract

**Background:** There is limited data evaluating the prescription practices for antithrombotic therapy in patients with atrial fibrillation (AF) following elective percutaneous coronary intervention (PCI).

**Objective:** This single-center, single-operator, retrospective cohort study aimed to evaluate trends of antithrombotic treatment strategies in patients with AF undergoing elective PCI.

**Methods:** Patients with AF who electively underwent PCI performed by a single interventionalist between April 2013 and May 2018 were identified. The primary outcome was the antithrombotic therapy at discharge assessed by chart review: triple (TAT, triple antithrombotic therapy) or dual (DAT, dual antithrombotic therapy) antithrombotic therapy and vitamin K antagonist (VKA) or non-vitamin K antagonist oral anticoagulant (NOAC), respectively.

**Results:** Of 6,135 screened patients, 259 met the inclusion criteria. Among these, 133 (51%) patients received NOAC- and 126 (49%) VKA-therapy. Compared with patients on NOAC therapy, patients treated with VKA had higher bleeding risk (mean HAS-BLED-Score; 2.3 vs. 2.0; *p* = 0.02) and more co-morbidities (estimated glomerular filtration rate <30 ml/min, 11 vs. 4%; *p* = 0.04; diabetes mellitus, 33 vs. 20%; *p* = 0.03; history of previous PCI, 37 vs. 21%; *p* < 0.01). TAT was prescribed more frequently if the prescription included VKA compared with NOAC (61 vs. 41%; *p* < 0.01). Prescription of TAT and VKA decreased throughout the observed period (2013: 100% vs. 2018: 6%; *p* < 0.01 and 2013: 91% vs. 2018: 28%; *p* < 0.01).

**Conclusion:** These observational data from a single center registry show a decrease of TAT- and VKA- prescription in favor of DAT with NOAC. Whether these observations are consistent with national or global trends should to be evaluated in further studies.

## Introduction

Atrial fibrillation (AF) increases the risk of stroke, embolization, and death (1). Oral anticoagulation (OAC) reduces this risk up to two-thirds irrespective of baseline risk (2). Non-vitamin K antagonist oral anticoagulants (NOACs) are safer and equally effective compared with vitamin K antagonist (VKA) (3).

The antithrombotic treatment of patients with AF undergoing elective percutaneous coronary intervention (PCI) poses a dilemma. Guidelines recommend acetylsalicylic acid (ASA) and clopidogrel following the placement of a coronary artery stent to an established OAC therapy, NOAC or VKA respectively hence forming a triple antithrombotic therapy (TAT) (4). However, studies show, that TAT results in an at least 2- to 3-fold increase in bleeding risk (5).

A recent network meta-analysis demonstrated that in patients with AF, TAT with VKA plus dual antiplatelet therapy should be avoided, whereas the use of a NOAC plus P2Y12 inhibitor (dual antithrombotic therapy [DAT]), without aspirin, should be the preferred treatment in patients with indication for OAC undergoing coronary stent implantation (6). The most recent consensus document recommends that TAT should be as short as possible or even avoided based on the individual's ischemic and bleeding risk (7).

The time course of uptake and implementation of evidence from key clinical trials and practice guidelines into everyday practice is crucial for patient safety (8).

This retrospective, observational study aimed to describe, in a single-center, single-operator, register analysis changes in antithrombotic management of AF patients post elective PCI in stable coronary artery disease (CAD). A second objective was to provide real world data on outcomes in terms of bleeding, re-hospitalization, and ischemic stroke under established therapy.

## Methods

### Cohort

In this single-center, retrospective cohort study we screened patients admitted to the ward 2a/b at Heart Center Freiburg University in-between April 2013 and May 2018 for elective coronary angiography with pre-existing or initial-diagnose of paroxysmal, persistent, or permanent AF and consecutive indication for OAC based on CHA2DS2-VASc score (4). Screening was conducted using in-house International Statistical Classification of Diseases and Related Health Problems (ICD)—coding. The protocol of this study conforms to the ethical guidelines of the 1975 Declaration of Helsinki. Data collection was part of the routine quality control program of our facility.

### Patient Characteristics

All patient clinical characteristics were obtained retrospectively from the electronic health record. Pharmacologic therapy before and after PCI was conducted according to the European Society of Cardiology (ESC) Guidelines. Laboratory values, such as creatinkinase were acquired within 24 h of the PCI. Patients were grouped according to the recommended antithrombotic therapy at discharge: TAT involving VKA or NOAC, respectively.

### Primary Percutaneous Coronary Intervention (PCI)

All patients received ASA (minimum of 250 mg followed by 100 mg/d if not already part of the permanent medication) and an adenosine-diphosphat (ADP) receptor blocker (Clopidogrel 600 mg directly before PCI followed by continuous intake of 75 mg per day). Unfractionated heparin (5,000 U) was administered prior to angiography. The same physician carried out all PCIs. The majority of the procedures were performed by radial access. All patients received new-generation drug-eluting stents (DES).

### Outcome

The primary outcome was the antithrombotic therapy at discharge assessed by chart review: triple (TAT, triple antithrombotic therapy) or dual (DAT, dual antithrombotic therapy) antithrombotic therapy and VKA or NOAC, respectively. Secondary outcomes were rehospitalisation, death, bleeding requiring medical attention, and stroke.

After the index-PCI patients were followed-up for a total of 3 years by the in-house quality management. Follow-up included questionnaires as well as telephone-contact with physicians, patients, and clinics 3 months, 1 year, and 3 years after PCI.

### Definitions

The bleeding type was characterized using the consensus reached in 2011 by the “Bleeding Academic Research Consortium” (BARC) (9). We calculated the CHA2DS2-VASC score to assess stroke risk. We calculated the HAS-BLED score bleeding risk score, wherein a score of ≥ 3 indicates high risk. The value “labile International Normalized Ratio (INR)” in HAS-Bled score was set to zero in NOAC patients.

### Statistics

Continuous patient data were compared using a *T*-test, if found to follow a Gaussian distribution, otherwise data underwent a Mann–Whitney *U*-test. Categorical differences between patient groups were compared using a Chi-square analysis (Fisher's exact test). Continuous variables are presented as mean ± standard deviation (*SD*) if found to follow a Gaussian distribution according to the D'Agosstino-Pearson omnibus normality test, or as median with lower and upper quartiles if found to follow a non-Gaussian distribution. Categorical patient characteristics are presented as percentages.

A *p*-value of <0.05 was considered statistically significant for all analyses.

All analyses were performed using Graph Pad Prism Version 6.0 (Prism 6 for Mac OS X; GraphPad Software, Inc., La Jolla, CA).

## Results

Of 6,135 patients hospitalized for elective coronary angiography between 04/2013 and 05/2018, 259 (4.2%) were patients with AF under VKA or NOAC therapy that underwent PCI with additional anti-platelet therapy after discharge. Of those 126 were treated with VKA and 133 with NOAC (59% rivaroxaban; 33% apixaban; 3% edoxaban; 5% dabigatran). The median follow up was 401 days (IQR 171–1104) post PCI. The median age was 77 years (IQR 71–82). 34% of included patients were female (Table 1).

**Table 1:** Baseline characteristics of included patients.

	**NOAC**_****(***n*** = 133)****_		**VKA**_****(***n*** = 126)****_		***P***	**TAT**_****(***n*** = 132)****_		**DAT**_****(***n*** = 127)****_		***P***
	n^*^	%^*^	n^*^	%^*^		n^*^	%^*^	n^*^	%^*^
Hypertension	123	92.5	120	95.2	0.5	120	90.9	123	96.8	0.08
Diabetes mellitus	27	20.3	42	33.3	0.03	29	22.0	40	31.5	0.1
eGFR <30 ml/min	5	3.8	14	11.1	0.04	10	10.3	9	7.1	0.5
History of PCI	28	21.1	47	37.3	<0.01	40	30.3	35	27.6	0.7
History of bleeding	12	9.0	11	8.7	0.9	10	7.6	13	10.2	0.6
Heart failure (HF) (LVEF^**^ <41%)	32	24.1	30	23.8	0.9	31	23.5	31	24.4	0.9
HF LVEF 41–50%	29	21.1	19	15.1	0.2	17	12.9	31	24.4	0.03
Hypercholesterinaemia	79	59.4	75	59.5	0.9	71	53.4	83	65.4	0.07
Active/former smoking	43	32.3	39	30.9	0.9	52	39.4	32	25.2	0.02
Sex (female)	49	36.8	39	30.9	0.4	42	31.8	46	36.2	0.5
Paroxysmal AF	83	62.4	52	41.2	<0.01	61	46.2	75	59.1	0.05
Persistent AF	47	35.3	73	57.9	<0.01	69	52.3	46	36.2	0.09
Age (years)	77	72–82	77	70–81	0.6	77	70–81	77	72–83	0.3
eGFR (ml/min)	66	±18.6	56	±22.4	<0.01	63	±21.1	61	±20.5	0.4
Thrombocytes (thousand/ul)	212	178–269	198	159–249	<0.01	205	168–253	208	168–262	0.9
HAS-BLED score^***^	2.05	±0.65	2	2–3	0.02	2	2–2.7	2	2–2.2	0.8
CHA2DS2- VASc-score^****^	4.6	±1.4	5	4-5	0.4	4.6	±1.2	4.8	±1.7	0.2
Number of stents	1	1–2	2	1–2	0.4	2	1–2	1	1–2	0.1
Hemoglobin (g/dl)	13.6	±1.6	13.3	±2.1	0.3	13.7	±1.9	13.5	12–14.4	0.1

* *Everything above the line is number (n) in percent (%); everything below the line is median with Interquartile range (IQR) or mean with standard deviation (SD)*.

** *Left ventricular ejection fraction*.

*** *Mnemonic for hypertension, abnormal renal and liver function, stroke, bleeding, labile international normalized radio, elderly and drugs or alcohol*.

**** *Mnemonic for congestive heart failure, hypertension, age >75 years, diabetes mellitus or thromboembolism, vascular disease, age 65–74 years and Sex*.

### Oral Anticoagulation (Vitamin K- and NOAC-Therapy)

**Figure 1:**
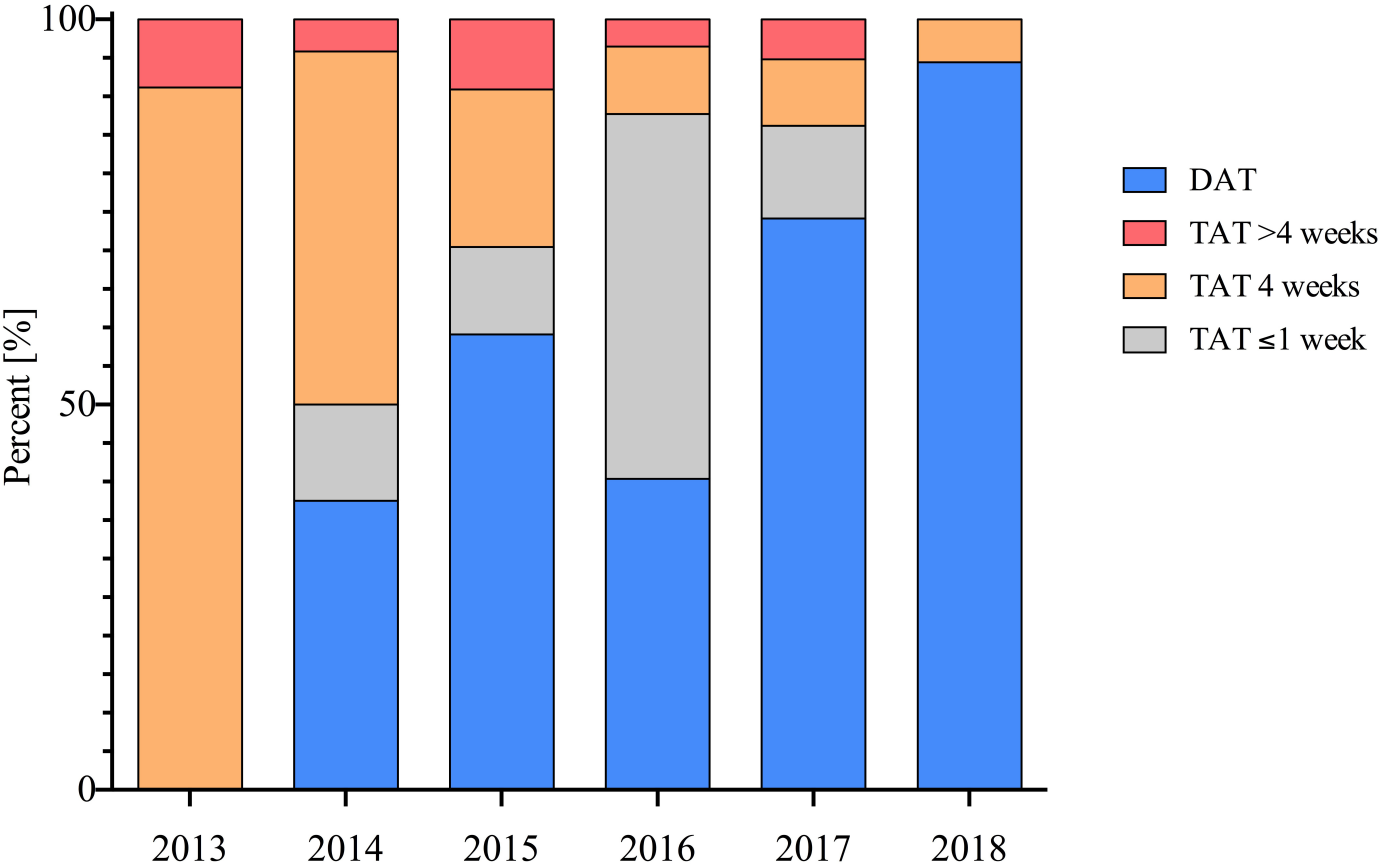
Implementation and duration of TAT and DAT in patients post PCI therapy throughout the observed period in percent (%).

The number of patients receiving NOAC-based therapy post PCI increased significantly from 2013 to 2018 (9 vs. 72%; *p* < 0.001) (Figure 1).

Compared with patients on NOAC therapy, patients prescribed with VKA had a higher bleeding risk profile (mean HAS-BLED score; 2.05 vs. 2.0 *p* = 0.02) and more co-morbidities (chronic kidney disease [CKD] [eGFR <30 ml/min] [11 vs. 4% *p* = 0.02]; diabetes mellitus [33 vs. 20% *p* = 0.02]; history of previous PCI [37 vs. 21%; *p* = 0.006]). Patients with NOAC therapy had higher platelet count at inclusion (212 thousand [IQR 178–269] vs. 198 thousand [159–249]; *p* = 0.009) (Table 1).

Prescription rates of OAC varied depending on episode timing and termination of AF as categorized by American College of Cardiology (ACC), the American heart Association (AHA) and the European Society of Cardiology (ESC) guidelines with patients with persistent AF receiving a prescription for VKA more often (58 vs. 35%; *p* < 0.001) and patients with paroxysmal AF receiving NOAC therapy more often (62 vs. 41%; *p* = 0.001). There was no statistically significant difference in CHA_2_DS_2_- VASc-score, or heart-failure (Table 1).

### Triple Antithrombotic Therapy

**Figure 2:**
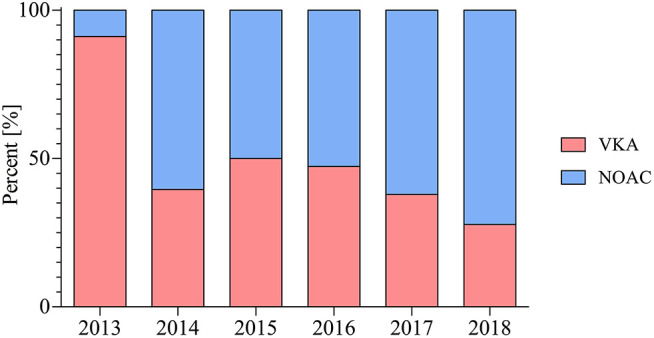
Choice of oral anticoagulation in patients post PCI therapy throughout the observed period in percent (%).

The prescription of TAT post PCI decreased significantly during the observed period from 2013 to 2018 (100 vs. 6%; *p* < 0.001) (Figure 2). More patients receiving VKA therapy also received TAT when compared with patients treated with NOAC (61 vs. 41%; *p* = 0.002).

Dropping ASA occurred earlier in NOAC-treated patients when compared with VKA-treated patients (after 1 week 42 vs. 22%; *p* = 0.025 / after 4 weeks 40 vs. 66%; *p* = 0.004).

Smokers or patients with history of smoking were more likely to receive TAT (39 vs. 25%; *p* = 0.02) (Table 1).

### Outcome

There was a statistically significant difference in between the length of follow-up (f/u) for the different groups. (f/u: TAT [543 (374–1115) days] vs. DAT [370 (43–742) days]; *p* = <0.001 and f/u: NOAC [378 (87–894) days] vs. VKA [473 (342–1111) days]; *p* = 0.003). Events for the different cohorts are described in Table 2.

**Table 2:** Secondary outcomes during follow-up.

	**NOAC**_****(***n*** = 133)****_		**VKA**_****(***n*** = 126)****_		**TAT**_****(***n*** = 132)****_		**DAT**_****(***n*** = 127)****_	
	***n***	**%**	***n***	**%**	***n***	**%**	***n***	**%**
Bleeding Events (%)	9	6.8	9	7.1	9	6.8	9	7.1
Type 2^*^	5	3.7	3	2.4	3	2.2	5	4.0
Type 3A^*^	2	1.5	2	1.6	3	2.2	1	0.8
Type 3B^*^	1	0.8	1	0.8	1	0.8	1	0.8
Type 3C^*^	0	0.0	1	0.8	1	0.8	0	0.0
Type 5^*^	1	0.8	2	1.6	1	0.8	2	1.6
ISTH^**^ major bleeding	3	2.2	5	4.0	4	3.0	4	3.1
Ischemic stroke	3	2.2	5	4.0	4.0	3.0	4	3.1
Re-hospitalization	60	45.1	71	56.3	72	54.6	59	46.5
Re-PCI	18	13.5	23	18.2	22	16.7	19	15.0
Stent-thrombosis	1	0.8	0	0.9	1	0.8	0	0.0
Death	18	13.5	19	15.1	16	12.1	21	16.56

* *Bleeding Academic Research Consortium*.

** *International Society on Thrombosis and Haemostasis*.

## Discussion

In this single-center, single-operator, retrospective, observational registry we analyzed trends in prescription patterns of antithrombotic therapy in clinical everyday practice.

Prescription rate of NOAC based therapy post PCI for AF patients has increased significantly in the observed period from April 2013 to May 2018. This rise of NOAC therapy is fuelled by numerous limitations and challenges with the pre-existing therapy with VKA, which requires frequent monitoring and has numerous drug and dietary interactions, as well as an increased intracranial bleeding rate (10).

In antithrombotic combination therapy post PCI, large randomized clinical trials show that NOAC instead of VKA reduces bleeding, including major and intracranial hemorrhages (6).

In our study patients with higher HAS-BLED-Score received NOAC less often than VKA (11). This underutilization of NOAC might reflect individual concerns about bleeding in high risk patients. Conservative prescription patterns might prevail in clinical everyday practice, contrary to evidence.

In our population patients on VKA therapy had a significantly higher prevalence of previous PCI. This is in line with other studies showing a higher atherothrombotic risk profile in VKA patients (12).

Randomized trials have demonstrated that DAT with NOACs are safer compared to TAT with VKA (7). The current paradigm in antithrombotic management of AF patients post PCI is that TAT should be as short as possible or even avoided based on the individual's ischemic and bleeding risk profile (7). Accordingly, we show a decrease of prescription of TAT in included patients over the observed period (Figure 1).

While in 2013, when a consensus document connected AF with an increased atherothrombotic risk, TAT was ubiquitously applied; in 2018, as results of large RCTs suggest that TAT should be as short as possible or even avoided, merely a fraction of included patients received TAT (6, 13–15).

Nevertheless, there is still uncertainty which patients might benefit from TAT post PCI. Current guidelines recommend evaluating TAT in patients with high ischemic and low bleeding risk (7).

Our data indicate the uptake of guideline recommendations into clinical practice throughout the years. They highlight the uptake of NOACs in post PCI antithrombotic therapy. Our results also show that in elective PCI significantly more patients receiving VKA therapy also received TAT when compared with patients treated with NOAC. Dropping ASA in TAT treated patients occurred earlier in NOAC-treated patients when compared with VKA-treated patients. This might be partly owed to conservative prescription patterns of NOAC in TAT and due to the fact that NOAC therapy emerged in the later years of the observation period when guidelines recommend that TAT should be as short as possible or even avoided based on individual risk factors.

## Limitations

One main limitation is the retrospective character of this study. We included patients with stable disease, excluding patients with an acute coronary syndrome. The generalizability of this single-center registry is also limited as prescription of antithrombotic therapy in AF patients after PCI varies substantially among sites (16).

The patients with TAT regime were followed over a longer period than patients with a DAT.

Patients with VKA were followed over a longer period than patients with NOAC. The vast majority of included patients under NOAC-therapy where treated with rivaroxaban or apixaban therapy. This limits the generalizability of our results to other NOACs. We did not collect data on the process of shared decision making. Finally, since the treating interventionist decided on the therapy post PCI our results are subject to potential bias and confounding.

## Conclusion

These observational data from a single center suggest that the prescription pattern of antithrombotic therapy in AF patients after PCI changed over the past 6 years. Duration and prescription of TAT and VKA decreased in favor of DAT with NOAC. Patients treated with VKA had a higher bleeding risk profile at baseline and more co-morbidity. Whether these observations are consistent with global trends needs to be evaluated in further studies.

## Data Availability Statement

The data of this study is available on reasonable request to the corresponding author.

## Ethics Statement

The protocol of this study conforms to the ethical guidelines of the 1975 Declaration of Helsinki and was henceforth approved by the institutional ethical committee of University of Freiburg (permit numbers EK345/20).

## Disclosure

CO reports research support from the German Research Foundation and speaker honoraria from Bayer Vital GmbH. MM reports receiving speakers fees from Bayer, Bristol-Myers-Squibb, Pfizer, Boehringer-Ingelheim, and Daichii-Sankyo.

## Author Contributions

MM, CO, and LH designed the study. LH and MD collected the data. MM, LH, MD, CO, DD, MH, and CB analyzed the data. LH, CO, and MM wrote the manuscript. MM performed all invasive procedures. All authors contributed to the article and approved the submitted version.

### Conflict of Interest

The authors declare that the research was conducted in the absence of any commercial or financial relationships that could be construed as a potential conflict of interest.

## Abbreviations

PCI: Percutaneous coronary intervention
AF: Atrial fibrillation
VKA: Vitamin k-antagonists
NOAC: Non-vitamin an antagonist oral anticoagulant
OAC: Oral anticoagulation
CAD: Coronary artery disease
DES: Drug-eluting stents
BMS: Bare metal stents
ASA: Acetylsalicylic acid
DAPT: Dual anti-platelet therapy
TAT: Triple antithrombotic therapy
DAT: Dual antithrombotic therapy.
