# Supplementary figures and images for: Dual-Pathway Antithrombotic Therapy in Patients With Atrial Fibrillation After Percutaneous Coronary Intervention in Stable Coronary Artery Disease: A Single-Center, Single-Operator, Retrospective Cohort Study
Source: Front Med (Lausanne). 2020 Sep 30;7:414. doi: 10.3389/fmed.2020.00414 (PMC7561383; doi:10.3389/fmed.2020.00414)

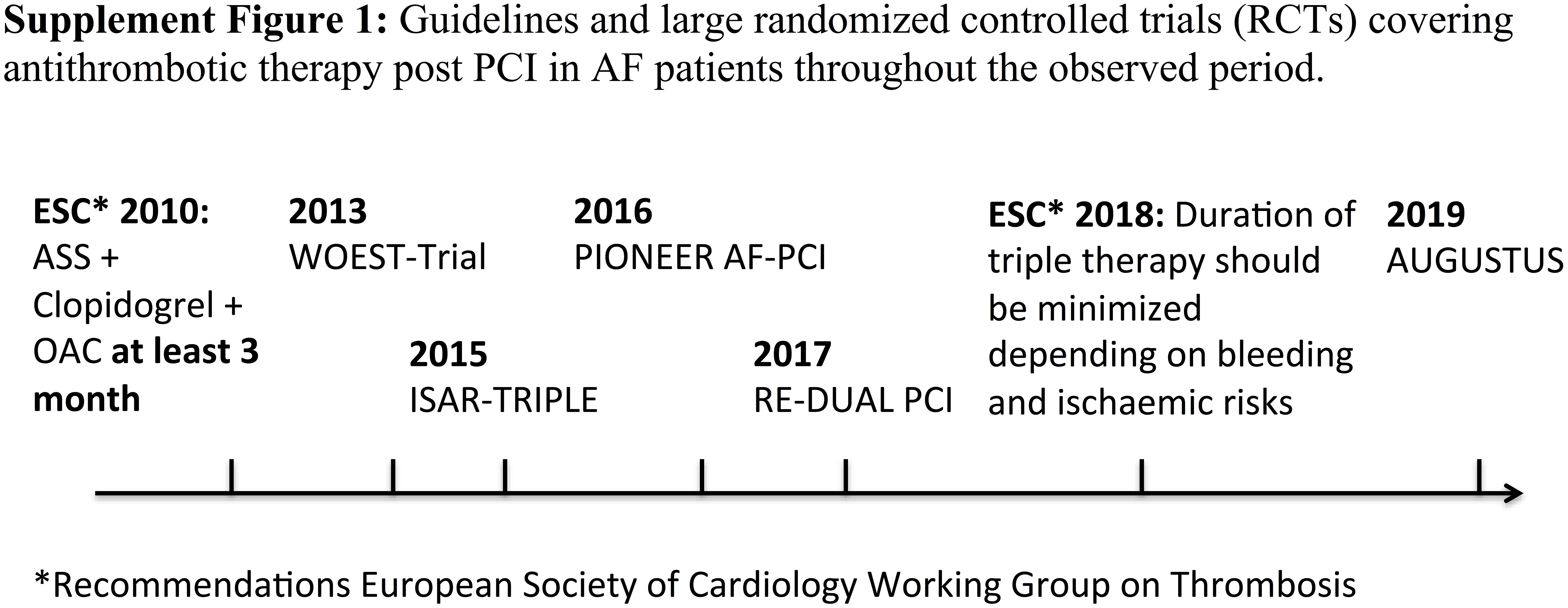

Supplement: Supplementary file 1 [file Image_1.JPEG]
